# Dural Ectopic Lymphatic Structures Accumulate During Aging and are Dysregulated in Neurodegenerative Diseases

**DOI:** 10.1002/alz70855_096532

**Published:** 2025-12-23

**Authors:** Amit Fruitman Davidi, Sophie Shirenova, Karin Vardy, Giulio Benedetti, Dolev Michaelovitch, Ravit Madar, Eitan Okun

**Affiliations:** ^1^ Bar‐Ilan University, Ramat‐Gan, Israel; ^2^ Bar‐Ilan University, Ramat‐Gan, Ramat‐Gan, Israel

## Abstract

**Background:**

This study investigates the formation and role of dural ectopic lymphoid structures (ELS) in the context of aging and neurodegenerative diseases, as these structures could serve as potential mediators of neuroimmune interactions and therapeutic targets. Using mouse models of early‐onset Alzheimer's disease (EOAD), tauopathy, and Down syndrome (DS), the study examines ELS accumulation, complexity, and interactions with brain pathology to illuminate their role in disease mechanisms.

**Method:**

Cranial dural meninges from aged wild‐type and transgenic mice (5xFAD and APP/PS1 for EOAD, K257T/P301S for tauopathy, and Dp1Tyb for DS) were analyzed. ELS were characterized by immunofluorescence using markers such as CD45R (B cells) and CD3 (T cells). Correlations between ELS dynamics and amyloid or tau pathology were assessed.

**Result:**

Meningeal ELS accumulate with age in wild‐type mice, showing sex‐specific patterns. Male mice exhibited a continuous increase in ELS with age, while female mice peaked at 12 months, possibly due to age‐related estrogen decline and its immunomodulatory effects. In 5xFAD mice, ELS formation intensified with pathology, whereas APP/PS1 mice displayed reduced ELS numbers with aging. Tauopathy and DS models showed significantly fewer ELS than controls. Correlations between ELS complexity and amyloid pathology varied by model, reflecting differences in how ELS relate to amyloid burden within distinct pathological contexts.

**Conclusion:**

Dural ELS exhibit age‐, sex‐, and pathology‐specific dynamics, reflecting their role in CNS pathology. Their differential regulation in AD, tauopathy, and DS highlights ELS as key players in the neuroimmune interface. Future research could explore manipulating ELS formation or function to mitigate neurodegenerative processes, potentially guiding immunomodulatory therapies tailored to specific diseases or patient populations.

